# High CD49a+ NK cell infiltrate is associated with poor clinical outcomes in Hepatocellular Carcinoma

**DOI:** 10.1016/j.heliyon.2023.e22680

**Published:** 2023-11-21

**Authors:** Alessandra Zecca, Valeria Barili, Carolina Boni, Paola Fisicaro, Andrea Vecchi, Marzia Rossi, Valentina Reverberi, Anna Montali, Giuseppe Pedrazzi, Carlo Ferrari, Elisabetta Cariani, Gabriele Missale

**Affiliations:** a Unit of Infectious Diseases and Hepatology, Laboratory of Viral Immunopathology, Azienda Ospedaliero–Universitaria of Parma, 43126 Parma, Italy; b Department of Medicine and Surgery, University of Parma, 43126 Parma, Italy; c Independent Researcher, Italy

## Abstract

NK cells infiltrating Hepatocellular Carcinoma (HCC) may express residency markers such as Integrin Subunit Alpha 1 (CD49a) that have been associated with nurturing functions in the decidua, and characterized by the production of angiogenic factors as well as loss of cytotoxicity. CIBERSORT, a computational analysis method for quantifying cell fractions from bulk tissue gene expression profiles, was used to estimate the infiltrating immune cell composition of the tumor microenvironment from gene expression profiles of a large cohort of 225 HCCs in the public GEO database. Decidual-like CD49a+ NK cells, in addition to another 22 immune cell populations, were characterized and thoroughly investigated so that HCC cell heterogeneity in a large cohort of 225 HCCs from the public GEO database could be studied. An inverse correlation of the expression of CD49a+ NK-cells and CD8^+^ T-cells suggested a negative association with clinical outcomes. This result was confirmed in a further validation cohort of 100 HCC patients from The Cancer Genome Atlas, Liver Hepatocellular Carcinoma (TCGA-LIHC). Cox regression analysis did not identify CD49a+ cells as a variable independently associated with survival. However, a more abundant infiltrate of this subset was present in patients at a more advanced pathological and clinical HCC stage. In conclusion, we found that NK cells, with a decidual-like gene expression profile, are enriched in HCC, and their abundance increases not only in tumor size but also at advanced stages of the disease suggesting that these cells play a role in tumor growth. For this reason, these NK cells may represent a possible new target for immunotherapeutic approaches in HCC.

## 1 Introduction

Natural killer (NK) cells are the main effectors of innate immunity. Circulating NK cells are generally classified according to the intensity of CD56 expression. CD56^BRIGHT^ and CD56^DIM^ NK-cell populations feature functional differences in terms of cytotoxicity and cytokine production; CD56^DIM^, which represents 90% of circulating NK cells, is considered more cytotoxic; CD56^BRIGHT^ are more prone to secrete interferon-γ (IFN-γ) and tumor necrosis factor-α (TNF-α), though they can readily acquire effector functions upon cytokines activation [1]. In tissue, NK cells may be present at a high concentration compared to other lymphomononuclear cells as shown in the liver and uterus where NK cells represent 50–70 % of all the infiltrating lymphocytes [[2], [3], [4]]. Tissue-resident NK cells are mainly CD56^BRIGHT^ and express different integrins and chemokine receptors such as CD49a, CD69, CD103, CD11b, and CXCR6. Tissue-resident NK cells (trNK-cells) may diverge significantly from circulating conventional NK cells (cNK-cells) by exhibiting not only effector activity against viruses and transformed cells but also regulatory and trophic functions in different physiologic conditions and diseases. Liver-infiltrating CXCR6+ NK cells account for more than 50% of the infiltrating Natural Killer cells compared to cNK-cells that express this phenotype at a low frequency. CXCR6 positive NK cells show different expressions of T-Box Transcription Factor 21 (Tbet) and Eomesodermin (Eomes) transcription factors in the periphery and tissues being mainly Tbet^hi^ Eomes^lo^ in cNK-cells and Tbet^lo^ Eomes^hi^ in the liver [4,5]. CD49a is an adhesion molecule that in mice identifies liver resident NK cells as CD49a+ DX5− [6] while circulating NK cells are CD49a−DX5+. These trNK-cells depend for their differentiation on Tbet and not Eomes, showing similarities with class I Innate lymphoid cell - ILC1 [6,7]. CD49a+ DX5− trNK cells were also found in higher frequency in the uterus and other tissues, such as skin and adipose tissues [8]. However, in this case, trNK-cells are Eomes^hi^ [8].

In hepatocellular carcinoma, the frequency of NK cells in the tumor microenvironment is significantly reduced compared to the surrounding non-tumor tissue. Its quantity and quality are correlated with clinical outcomes after surgical treatment [[9], [10], [11]]. HCC infiltrating NK cells were found functionally deficient in previous studies [[9], [10], [11], [12], [13]]. In HCC, a higher frequency of CD49a+ NK cells was shown compared to the surrounding non-tumorous liver tissue and also to normal control livers. In particular, there was a significantly higher frequency of CD49a+ Eomes^hi^ NK-cells. These features resemble the NK phenotype which is similar to the abundant NK cell infiltrate of the uterus [8,14]. Decidual NK cells produce angiogenic factors and proteases for vascular growth, tissue remodeling, and support to placental development which plays a central role in successful pregnancies.

NK cells that produce angiogenic factors may support tumor expansion rather than control its growth. Indeed, a negative outcome associated with CD49a+ NK-cells infiltrating in HCC was previously reported [15].

Interest in the study of a tumor-immune microenvironment greatly increased with the advent of immunotherapy for several malignancies. Anti-VEGF and anti-PD-L1 represent first-line treatments for unresectable HCC with an objective response rate (ORR) of nearly 30% [16]. Even though response predictors in patients undergoing immunotherapy must be better defined, tumor mutational burden, neoantigens, and tumor-infiltrating lymphocytes are promising response predictors [17]. To this end, in silico screening of data from single-cell and bulk tissue was applied using different methodological approaches to estimate immune composition and the relative amount of tumor-infiltrating immune cells [18]. Cell-type Identification by Estimating Relative Subsets Of RNA Transcripts (CIBERSORT) [19] is a machine learning-based methodology that infers relative frequencies of different immune cells from bulk gene-expression profiling data obtained from microarray or RNA-sequencing. By applying CIBERSORT to gene expression profiling data derived from decidual NK-cells (CD3^−^CD56^+^CD49a+ CD49b−) and circulating NK-cells (CD3^−^CD56^+^CD49a−CD49b+) [20], we generated a signature matrix to estimate the expression of CD49a+ cells in HCCs. Here, we applied the CD49a signature matrix to gene expression datasets of 225 hepatocellular carcinomas from GSE14520 as a training set, and 100 HCCs from TCGA-LIHC as validation cohort to evaluate a possible relation of CD49a + NK-cells within the tumor and the patient clinical outcome.

## 2 Methods

### 2.1 Gene expression data

Gene expression datasets obtained from Affymetrix microarray with corresponding clinical information of HCC were downloaded from Gene Expression Omnibus (GEO) GSE14520. Similarly, gene-expression data from RNA-seq were available from the LIHC cohort of The Cancer Genome Atlas (TCGA: https://portal.gdc.cancer.gov/).

### 2.2 Analysis of tumor-infiltrating immune cells

CIBERSORT, an analytical and machine learning-based tool developed by Newman et al. [21], estimates the abundance of cell types in a heterogeneous cell population from their RNA gene-expression profiles. CIBERSORT requires two input files: a “mixture file” that contains one or more gene expression profiles (GEPs), and a “signature matrix” which consists of a reference matrix gene-expression signature to estimate the relative proportions of each interested cell type. Transcriptome data were analyzed by CIBERSORTx, which is an improved CIBERSORT computational framework according to the procedure described by Steen CB et al. [22]. We first created a custom signature matrix from microarray data from manually-downloaded GSE97217 (Fu B, Immunity 2017), relating to differentially expressed genes (DEGs) of the comparison of sorted CD56^+^CD3^−^CD45^+^ CD49a+ CD49b- and CD56^+^CD3^−^CD45^+^ CD49a-CD49b+ cells. These populations were derived from fresh deciduas from the first trimester (CD49a+) and the peripheral blood of healthy donors (CD49b+) [20]. In particular, the custom signature matrix was created from GSE97217 gene expression data with the specific analysis module “Create Signature Matrix” of CIBERSORTx which required an input file with no redundant gene names and genes without expression level at zero, and formatted according to the default parameters of the application software [22]. Next, we used the previously published microarray-derived signature matrix for profiling 22 functionally defined human immune cell types, called LM22 [21] to evaluate the relative proportions of 22 types of tumor-infiltrating immune cells (TIICs) in the same patients. LM22 encompasses 547 genes that differentiate 22 human hematopoietic cell types, inclusive of various B- and T-cell types, plasma cells, NK-cells, and myeloid subsets. Specifically, the CIBERSORTx website provides an R script to convert Affymetrix raw files from GSE14520 data into a tabular format that is ready for the next CIBERSORT analysis. As requested, the converted file was then checked for the subsequent analysis in which the gene expression data could not be in a logarithmic scale without any redundant gene symbol, and then uploaded for the “Cell Fraction analysis module” using the default software parameters.

### 2.3 Statistical analysis

The correlation between different proportions of distinct immune cells from the GSE14520 dataset was analyzed by applying Pearson's correlation coefficient. Proportions of 22 TIICs or CD49a+ cells, different tumor and clinical characteristics, were evaluated as parameters that could affect the overall survival by Kaplan-Meier curves compared with the log-rank test. Cox proportional hazards regression was used for multivariate analysis including only the parameters that turned out to be statistically significant (p < 0.05) in univariate analysis. We checked for multicollinearity examining the correlation coefficients and variance inflation factor (VIF) values. Immune cell proportions from the liver and tumor were compared using the Wilcoxon signed-rank test, while a comparison of immune cell proportions in different clinical tumor stages was done with the Mann-Whitney *U* test. The statistical significance was considered for values p < 0.05.

## 3 Results

### 3.1 Clinical characteristics of GSE14520 patients

The GSE14520 data set collects gene expression data by Affymetrix microarray profiling of 225 hepatocellular carcinomas and of the corresponding non-tumorous livers obtained by surgical resections. Clinical data available for this cohort are represented in Table 1. The majority of patients are in BCLC-A or TNM stage I with more than 90% of them positive for HBV serum markers and almost a quarter actively replicating HBV. The majority of cases showed small-size uninodular tumors with alpha-fetoprotein levels below 50 U/L in more than 50% of the cases. At 24 months, 75% of the patients were alive.

**Table 1:** Demographic and clinical characteristics of patients. NA: not available; AVR-CC: active viral replication-chronic carrier.

	GSE14520	TCGA-LIHC
	*225 pts*	*100 pts*
***Age (median*** ± ***SE)***	*50* *±* *0.71*	*61* *±* *1.35*
***Gender (%)***		
*Male*	*191 (84.9 %)*	*66*
*Female*	*34 (15.1 %)*	*34*
***Pathologic staging***		
*I*	*93 (41.3 %)*	*50*
*II*	*77 (34.2 %)*	*17*
*III*	*49 (21.8 %)*	*22*
*IV*	*0 (0 %)*	*4*
*NA*	*6 (2.7 %)*	*7*
***Cirrhosis***		
*Yes*	*203 (90.2 %)*	
*No*	*22 (9.8 %)*	
***Multinodular***		
*Yes*	*45 (20 %)*	
*No*	*176 (78.2 %)*	
*NA*	*4 (1.8 %)*	
***AFP (>/<*300 ng/ml*)***		
*high*	*100 (44.4 %)*	
*low*	*118 (52.5 %)*	
*NA*	*7 (3.1 %)*	
***BCLC***		
*A*	*148 (65.8 %)*	
*B*	*22 (9.8 %)*	
*C*	*29 (12.9 %)*	
*NA*	*26 (11.5 %)*	
***HBV viral status***		
*neg*	*6 (2.7 %)*	
*AVR-CC*	*56 (24.9 %)*	
*CC*	*156 (69.3 %)*	

### 3.2 Clinical characteristics of the validation cohort

One hundred additional HCCs from the TCGA-LIHC database were randomly selected. These HCCs samples represent a further validation cohort. The retrieved mRNA gene expression data of this sample cohort was generated by RNAseq.

TNM and clinical staging were typical of early HCC, even though three patients presented metastasis (M1) and two cases were with locoregional lymph node dissemination, altogether four patients were in stage IV (Table 1). TCGA patient cohort median survival was 33.5 months.

Overall survival of the GSE14520 and cohort from TCGA-LIHC was significantly different with better survival for GSE14520 patients (p < 0.001. Log Rank test - data not shown).

### 3.3 Comparative expression of infiltrating lymphocyte subsets in tumor and non-tumor liver tissue

A comparison of the relative representation of the Tumor-infiltrating immune cells (TIICs) showed the presence of 22 immune cell subsets and CD49a positive NK-cell subset in the two compartments of GSE14520 cohort (Table 2). A significant difference was revealed for almost all the subsets with distinctive enrichment of immune cells in the liver. The more dominant immune subsets in the tumor were: the naïve CD4 T cells, regulatory T cells, activated dendritic cells, resting mast cells, M0 macrophages, and CD49a NK cells (Table 2).

**Table 2:** CIBERSORT expression values in the tumor and surrounding non-tumor tissue.

	TUMORS (225)			NON-TUMORS (220)				
	Mean	Std. Deviation	Std. Mean Error	Mean	Std. Deviation	Std. Error of Mean	delta non-Tumors/Tumors	P value
**T cells CD8**	0.092	0.061	0.004	0.121	0.051	0.003	0.028	***<0.0001***
**Macrophages M1**	0.127	0.034	0.002	0.149	0.038	0.003	0.022	***<0.0001***
**B cells naive**	0.079	0.027	0.002	0.101	0.033	0.002	0.021	***<0.0001***
**Mast cells activated**	0.022	0.025	0.002	0.043	0.040	0.003	0.021	***<0.0001***
**T cells gamma delta**	0.043	0.033	0.002	0.063	0.044	0.003	0.020	***<0.0001***
**Monocytes**	0.022	0.020	0.001	0.033	0.025	0.002	0.012	***<0.0001***
**Dendritic cells resting**	0.050	0.021	0.001	0.057	0.022	0.001	0.006	***0.0073***
**Plasma cells**	0.010	0.019	0.001	0.015	0.018	0.001	0.005	***<0.0001***
**Macrophages M2**	0.084	0.035	0.002	0.086	0.040	0.003	0.002	0.5994
**NK cells resting**	0.009	0.017	0.001	0.011	0.019	0.001	0.002	0.3387
**Eosinophils**	0.002	0.004	0.000	0.002	0.004	0.000	0.000	0.9262
**B cells memory**	0.004	0.011	0.001	0.003	0.012	0.001	0.001	***0.0049***
**Neutrophils**	0.018	0.014	0.001	0.016	0.011	0.001	0.002	0.193
**T cells CD4 memory activated**	0.010	0.019	0.001	0.005	0.010	0.001	0.005	***0.0004***
**T cells follicular helper**	0.056	0.023	0.002	0.051	0.020	0.001	0.006	***0.0199***
**T cells CD4 memory resting**	0.108	0.070	0.005	0.102	0.059	0.004	−0.006	0.3316
**NK cells activated**	0.057	0.032	0.002	0.050	0.030	0.002	−0.007	0.0535
**T cells CD4 naive**	0.019	0.025	0.002	0.012	0.024	0.002	−0.007	***<0.0001***
**T cells regulatory (Tregs)**	0.025	0.021	0.001	0.017	0.017	0.001	−0.008	***<0.0001***
**Dendritic cells activated**	0.015	0.020	0.001	0.006	0.011	0.001	−0.009	***<0.0001***
**Mast cells resting**	0.043	0.044	0.003	0.025	0.028	0.002	−0.018	***<0.0001***
**Macrophages M0**	0.104	0.073	0.005	0.032	0.026	0.002	−0.072	***<0.0001***
**NK CD49a+**	0.735	0.119	0.008	0.640	0.104	0.007	−0.095	***<0.0001***

### 3.4 Correlation matrix of 22 TIICs and CD49a+ NK-cells subset in the tumors

**Fig. 1:**
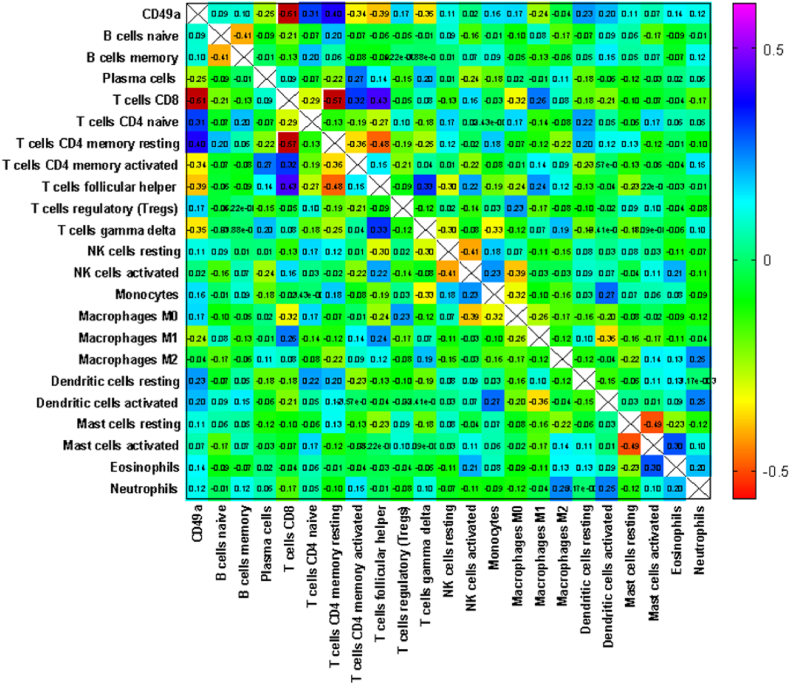
Correlation of the immune cell populations infiltrating HCCs in the GSE14520 cohort. Pearson's correlation matrix: colors represent Pearson's ‘r’ values, ranging from −1.00 to 1.00 from indirect to direct correlation.

Then, we generated a Pearson's correlation matrix of the relative expression of the different immune subsets derived from the GSE14520 patient data. By setting the correlation coefficient cut-off at 0.5 or −0.5, we could identify only two significant relations: the indirect correlation between CD49a positive NK-cells and CD8 T-cells, and between memory resting CD4 and CD8 T cells (Fig. 1). In lowering the threshold of the correlation coefficient to 0.3 or −0.3, we found several other significant correlations. A direct correlation with naïve CD4 and memory-resting CD4 T-cells and an indirect correlation with memory-activated CD4 T-cells, follicular helper T-cells, and the gamma delta T-cells were evident when we focused on CD49a NK-cells.

### 3.5 Prognostic significance of clinical and immunologic parameters

The survival status of patients was evaluated by taking the clinical characteristics and the relative expression of tumor-infiltrating immune cells into consideration.

**Fig. 2:**
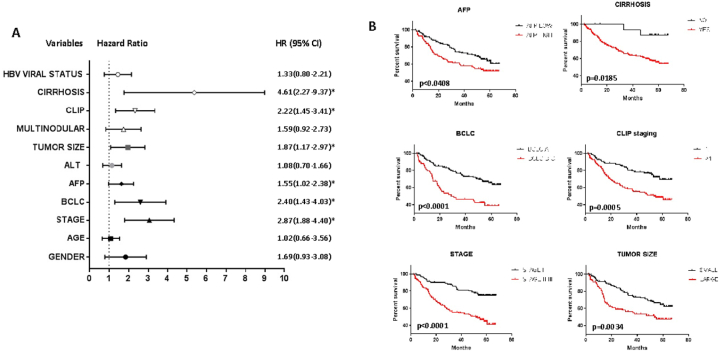
GSE14520 patients survival according to different demographic, clinical, and pathological variables. A. Forest plot showing hazard ratios (HR) and confidence intervals of the survival statistics. *statistically significant hazard ratios. Active viral replication and chronic carrier state are the two categories of HBV viral status. Cut-off values were: cirrhosis (yes or not), CLIP (0 + I and > I), Multinodular (yes or not), tumor size (>< 5 cm) ALT (<> 50UI/ml), AFP (<> 300 ng/ml), BCLC (0 + A and B + C), stage refers to pathologic stage: the comparison is between stage I and combined stage II and III. B. Only survival curves of significant parameters are represented.

Fig. 2 shows the survival analysis according to the clinical parameters in the training set. Cox regression analysis identified TNM staging as an independent factor associated with the outcome while the cirrhosis status, alpha-fetoprotein, and staging by BCLC or CLIP did not reach statistical significance (Fig. 2 A). The presence of cirrhosis, the clinical stage (CLIP and BCLC), pathological stage, tumor size, and alpha-fetoprotein were significantly associated with a worse outcome (Fig. 2 B).

Gender, age, and the pathological stage in the validation cohort were analyzed which confirmed a significant association with a lower survival rate for the pathological stage (p < 0.05, HR = 1.81 [95 % CI = 1.00 to 3.1] not shown).

**Fig. 3:**
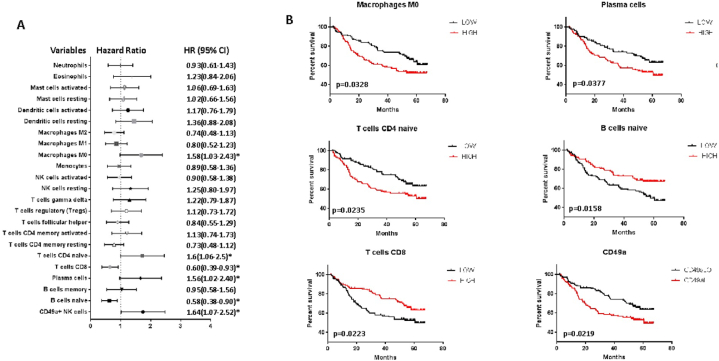
GSE14520 patient survival according to the relative expression of immune cell populations. A. Forest plot showing hazard ratios (HR) and confidence intervals of the survival statistics. *statistically significant hazard ratios. B. Survival curves of significant parameters are represented. Median values of expression of immune cell populations were used as cut-offs.

We then evaluated the association of the expression of all the different subsets derived from the CIBERSORTx analysis with the overall survival (Fig. 3 A). Macrophages M0, naïve CD4 T cells, plasma cells, and CD49a + NK-cells were all associated with an increased risk of death, while CD8 T cells and naïve B cells played a protective role (Fig. 3 B). Cox regression analysis indicated naïve B-cells as the independent protective subset. CD8 T-cells were also associated with protection (HR = 0.636 [95 % CI = 0.401 to 1.006]) even though they did not reach statistical significance (P = 0.053) (Fig. 5).

We also performed the multivariate analysis considering all statistically significant parameters either clinical or immunological. The Cox regression confirmed naïve B-cells and the pathological stage, and in this case, CD8 T-cells as well.

**Fig. 4:**
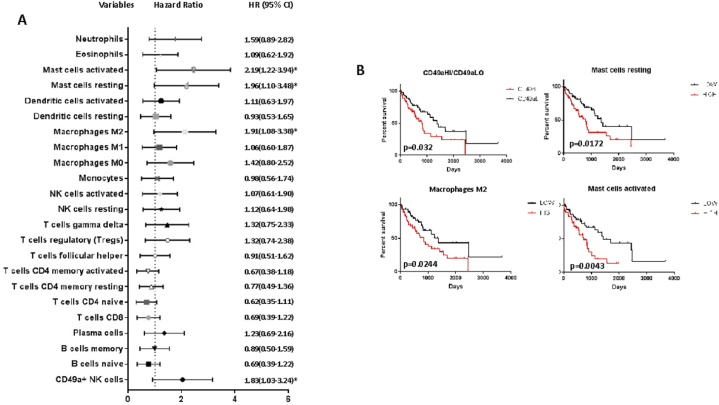
TCGA patient survival according to the relative expression of immune cell populations. A. Forest plot showing hazard ratios (HR) and confidence intervals of the survival statistics. *statistically significant hazard ratios. B. Survival curves of significant parameters are represented. Median values of expression of immune cell populations were used as cut-offs.

The prognostic relevance of immune cell expression levels was also analyzed in the TGCA validation cohort (Fig. 4 A and B). The univariate analysis confirmed CD49a positive NK-cells as a negative survival predictor as well as resting and activated mast cells and M2 macrophages (Fig. 4 A). The multivariate analysis, in this case, failed to identify a significant predictor (data not shown), possibly because the variables, in particular resting and activated mast cells, were highly correlated.

**Fig. 5:**
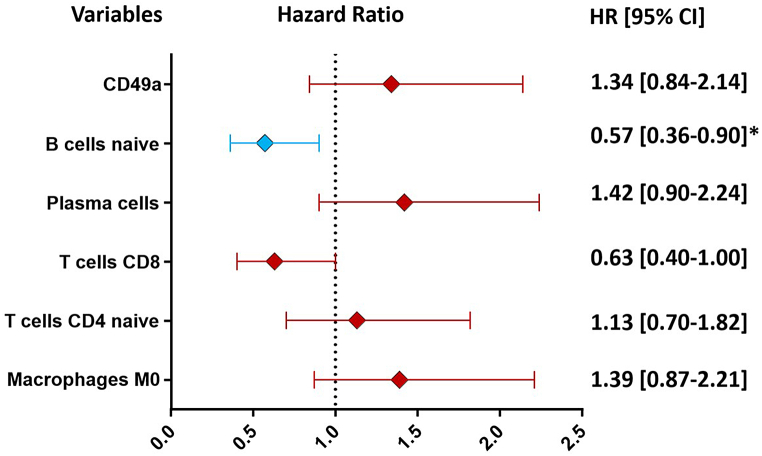
Multivariate analysis of immune cell populations from GSE14520, significantly associated with clinical outcome (OS). Forest plot shows hazard ratios (HR) and confidence intervals. *statistically significant hazard ratios.

### 3.6 CD49a-positive NK-cells are enriched in advanced-stage HCC

**Fig. 6:**
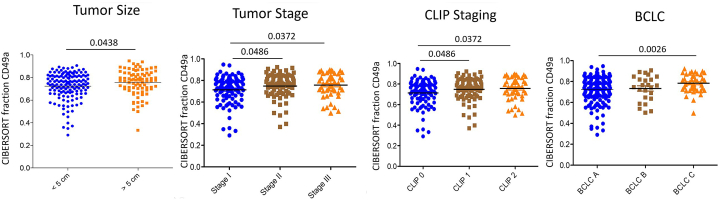
CD49a expression in GSE14520 patients HCCs according to tumor size, pathologic and clinical staging. Tumor size below or equal and above 5 cm diameter of the main HCC nodule, tumor stage according to American Joint Committee on Cancer (AJCC), The Cancer of the Liver Italian Program (CLIP) score, Barcelona Clinic Liver Cancer (BCLC).

Next, we evaluated the relative expression of the CD49a NK-cell subset in the different clinical and pathological stages. Significant differences were highlighted between pathological stages I, II, and III, BCLC A vs C, and between Clip stages. A significantly different distribution of CD49a expression was also present according to tumor size, as expected being part of staging process (Fig. 6). No differences could be identified according to ALT and alpha-fetoprotein levels, presence of cirrhosis, uninodular vs multinodular HCC, HBV replication status, age, and gender.

No significant difference in CD49a expression was found according to the different disease stages in the validation TGCA cohort.

## 4 Discussion

CD49a+ NK-cells represent 85% of decidual NK-cells [23]; they have reduced cytotoxic potential and produce pro-angiogenic factors with a nurturing key role for the fetus. We found an enrichment of CD49a+ Eomes^hi^ NK-cell phenotype in the tumor tissue of HCC patients. The enhanced prevalence of this phenotype might contribute to tumor growth and dissemination due to reduced cytotoxicity and production of VEGF and other angiogenic factors by NK cells [12].

In fact, enrichment of CD49a-positive NK cells in HCC was associated with worse clinical outcomes [15] in a previous study. We wanted to confirm and extend these results in this study.

CD49a expression was evaluated by the algorithm CIBERSORTx which deconvolutes the expression of a single-cell subset from a mixture of gene profiling generated by microarray or RNA-seq from whole tissue specimens. CD49a expression was measured in the HCCs and in the non-tumorous counterpart from 225 GSE14520-derived patient cohorts and other 100 TGCA-derived patients as the validation group.

While most of the immune cells were more prevalent in the liver than in the tumor counterpart, CD4 T-cells composed of regulatory T-cells, macrophages M0, and CD49a NK-cells were significantly more expressed in the tumor.

In performing a correlation analysis between the various types of tumor-infiltrating immune cells, we found a significant negative correlation between memory-resting CD4 T-cells and CD8 T-cells as well as between CD49a NK-cells and CD8 T-cells. CD49a NK-cells were also positively correlated with memory-resting CD4 T-cells and naïve CD4 T-cells. The prognostic meaning of different immune cells infiltrate maybe different according to the type of tumor, the tumor stage, sample size and methods of analysis. In any case, the meaning of CD8 and CD4 T-cell infiltrate has been reported with opposing direction [24], with good and dismal prognosis respectively. Thus, the indirect correlation of CD49a NK-cells and CD8 T-cells is strongly suggestive of an enrichment of these NK-cells in patients with worse clinical outcomes.

Then, we evaluated patient survival according to clinical parameters and immune cell infiltrate.

Pathological and clinical disease stages and alpha-fetoprotein were significantly associated with survival. Different immune cells were also associated with outcomes. In the GSE14520 cohort, the only two subsets showing a protective role were the CD8 T-cells and naïve B-cells, decreasing the risk of death by 40–42 %. In the TCGA, CD8 T-cells were associated with a positive outcome but did not reach statistical significance. Considering both cohorts, macrophages M0 and M2, mast cells, naïve CD4 T-cells, plasma cells, and CD49a NK-cells were all associated with an increased risk of death. These results agreed with previous studies conducted with similar methodological approaches [24,25] or other techniques [[26], [27], [28], [29]], while CD49a NK-cells had not previously been analyzed for their prognostic value in a large cohort of patients. In a previous study, Sun H. et al. [15] found a higher expression of checkpoint molecules on HCC infiltrating CD49a+ NK-cells in 28 patients undergoing liver resection, as well as an association with negative tumor characteristics such as portal neoplastic thrombosis or absence of tumor capsule, and finally with patient survival.

Nowadays, with the rapid development of new immunotherapeutic protocols in cancer, the prognostic role of immune subsets has become a hot issue in personalizing treatments. In point of fact, not all patients are responsive to immunotherapy and could benefit from a different first line of treatment. The analysis of the immune cell infiltrate is becoming not only a prognostic but also a predictive tool for immunotherapy [27]. This growing analytical technology for new and available patient series integrated with clinical information provides for several tumors to be validated before specific treatments are determined. As for HCC, tumors have been classified in terms of the amount and quality of immune cell infiltrate [26] that identifies prognostic signatures. However, there is not sufficient data available to predict immunotherapeutic treatment outcomes. HCC is a complex disease since it arises in different liver disease backgrounds; emerging data suggest different responses to treatments in different contexts [30].

We found a negative prognostic role of CD49a+ NK-cells. Interestingly, this subset accumulated in tumors of more advanced stage and nodules size suggests a different tumor immune microenvironment with disease progression that could be responsible for the differentiation of these cells because of a higher degree of hypoxia and production of TGF-beta [31].

The limitation of our study is that the patients were all surgically resected and in most cases in early-stage HCC. Therefore, our results has to be confirmed in patients candidate for systemic treatment.

In conclusion, we analyzed a training and validation set of more than 300 HCC patients with enrichment in CD49a+ NK-cells in the tumor associated with a worse clinical outcome and an advanced disease stage. This is in line with previous results showing the expression of negative checkpoint molecules in these NK cells [15] and our previous results showing a pro-angiogenic response of these cells [12]. The present study underlines the interest in this tumor-enriched NK-cell subset and identifies a new target for possible immunotherapeutic strategies that could interfere with their nurturing activity on tumor growth.

## Funding

**‘**This work was supported by a grant from HUNTER-Accelerator Award 22,794 (AIRC, CRUK, AECC) and FIL-Quota Incentivante” of University of Parma and co-sponsored by Fondazione Cariparma

## Data availability statement

Data is available at Gene Expression Omnibus (GEO) and LIHC cohort of The Cancer Genome Atlas.

## CRediT authorship contribution statement

**Alessandra Zecca:** Conceptualization, Data curation, Formal analysis. **Valeria Barili:** Conceptualization, Writing – original draft. **Carolina Boni:** Data curation, Formal analysis. **Paola Fisicaro:** Data curation, Formal analysis, Writing – original draft. **Andrea Vecchi:** Data curation, Formal analysis. **Marzia Rossi:** Data curation, Formal analysis. **Valentina Reverberi:** Data curation, Formal analysis, Writing – review & editing. **Anna Montali:** Data curation, Formal analysis, Writing – review & editing. **Giuseppe Pedrazzi:** Data curation, Formal analysis, Methodology. **Carlo Ferrari:** Conceptualization, Writing – original draft. **Elisabetta Cariani:** Conceptualization, Writing – original draft, Writing – review & editing. **Gabriele Missale:** Conceptualization, Data curation, Methodology, Writing – original draft, Writing – review & editing.

## Declaration of competing interest

The authors declare that they have no known competing financial interests or personal relationships that could have appeared to influence the work reported in this paper.
